# Case Report: Retrosternal parathyroid lipoadenoma in an obese male patient—diagnostic pitfalls and postoperative biphasic parathyroid hormone dynamics

**DOI:** 10.3389/fsurg.2026.1793999

**Published:** 2026-07-28

**Authors:** Jin-shen Hu, Tao Zuo, Jun Fu, Kang-le Kong, Yong Liu

**Affiliations:** 1 School of Medicine, Jianghan University, Wuhan, Hubei, China; 2 Department of Thoracic Surgery, The Central Hospital of Wuhan, Tongji Medical College, Huazhong University of Science and Technology, Wuhan, Hubei, China; 3 Department of Thoracic Surgery, Hubei Cancer Hospital, Affiliated to Huazhong University of Science and Technology, Wuhan, Hubei, China

**Keywords:** biphasic PTH, hypocalcemia, parathyroid lipoadenoma, retrosternal ectopia, subxiphoid VATS

## Abstract

**Introduction:**

Parathyroid lipoadenoma is a rare histologic variant of parathyroid adenoma. Its occurrence in the retrosternal mediastinum poses significant diagnostic and surgical challenges. The clinical significance lies not in its rarity, but in the diagnostic pitfalls it presents, particularly when located in the retrosternal mediastinum. We present a case that underscores the pitfalls in preoperative diagnosis and the intricacies of minimally invasive resection.

**Case presentation:**

A 36-year-old obese man (BMI 31 kg/m^2^, WHO Class I obesity) presented with an incidental anterior superior mediastinal mass. Preoperative computed tomography revealed a soft-tissue density lesion suspected to be a thymoma. The clinical implications of his recurrent nephrolithiasis and retrospectively identified albumin-corrected hypercalcemia (2.98 mmol/L, serum albumin: 46.5 g/L; reference range: 40–55 g/L) were overlooked. Given the patient's body habitus and the tumor's location cephalad to the innominate vein, subxiphoid video-assisted thoracoscopic surgery (VATS) under artificial pneumothorax was performed. Severe hypocalcemic tetany on postoperative day 1, followed by a characteristic biphasic parathyroid hormone (PTH) response, provided key diagnostic clues and ultimately led to a pathological diagnosis of parathyroid lipoadenoma.

**Conclusion:**

For patients with a mediastinal mass accompanied by hypercalcemia, comprehensive clinical evaluation is required to exclude ectopic primary hyperparathyroidism, rather than interpreting imaging findings in isolation. Subxiphoid VATS is the preferred technique for high retrosternal tumors in patients with challenging anatomical features. Familiarity with postoperative biphasic PTH changes and Hungry Bone Syndrome is essential for accurate interpretation of perioperative biochemical results.

## 1 Introduction

Parathyroid lipoadenoma is a benign neoplasm, constituting less than 1% of all parathyroid adenomas (1). It is characterized histologically by a prominent stromal component of mature adipose tissue, which must comprise more than 50% of the tumor volume (2). This distinctive pathology can occur in ectopic locations, with approximately 1%–2% of all parathyroid adenomas found in the mediastinum (3). The combination of an ectopic retrosternal location and lipoadenoma histology easily leads to misdiagnosis in clinical practice. We present a comprehensive case report of a retrosternal parathyroid lipoadenoma, where the diagnosis was established postoperatively following technically challenging subxiphoid video-assisted thoracoscopic surgery (VATS) resection.

## 2 Case presentation

A 36-year-old man (height: 170 cm, weight: 90 kg, body mass index 31 kg/m^2^, WHO Class I obesity) was admitted for an incidental anterior superior mediastinal mass discovered during a routine health screening. His history was significant for recurrent nephrolithiasis over ten years, requiring previous surgical intervention. The previous surgery was performed at an external hospital, and complete medical records—including stone composition analysis and historical measurements of serum calcium and parathyroid hormone (PTH)—were unavailable for preoperative assessment.

Physical examination revealed an obese body habitus with a short neck but no other remarkable findings. Cardiopulmonary, abdominal, neurological, and musculoskeletal examinations were unremarkable. There was no bone tenderness, limb numbness, muscle spasm, or other manifestations of hyperparathyroidism detected on physical examination.

The patient underwent routine health tests due to a family history of thymoma, as his father had a confirmed diagnosis of thymoma. The patient denied typical clinical manifestations of hyperparathyroidism, including fatigue, bone pain, polydipsia, and polyuria. The only suggestive clinical features were a more than 10-year history of recurrent nephrolithiasis requiring surgical intervention, together with retrospectively identified hypercalcemia.

### 2.1 Preoperative workup and diagnostic oversight

#### 2.1.1 Imaging

**Figure 1:**
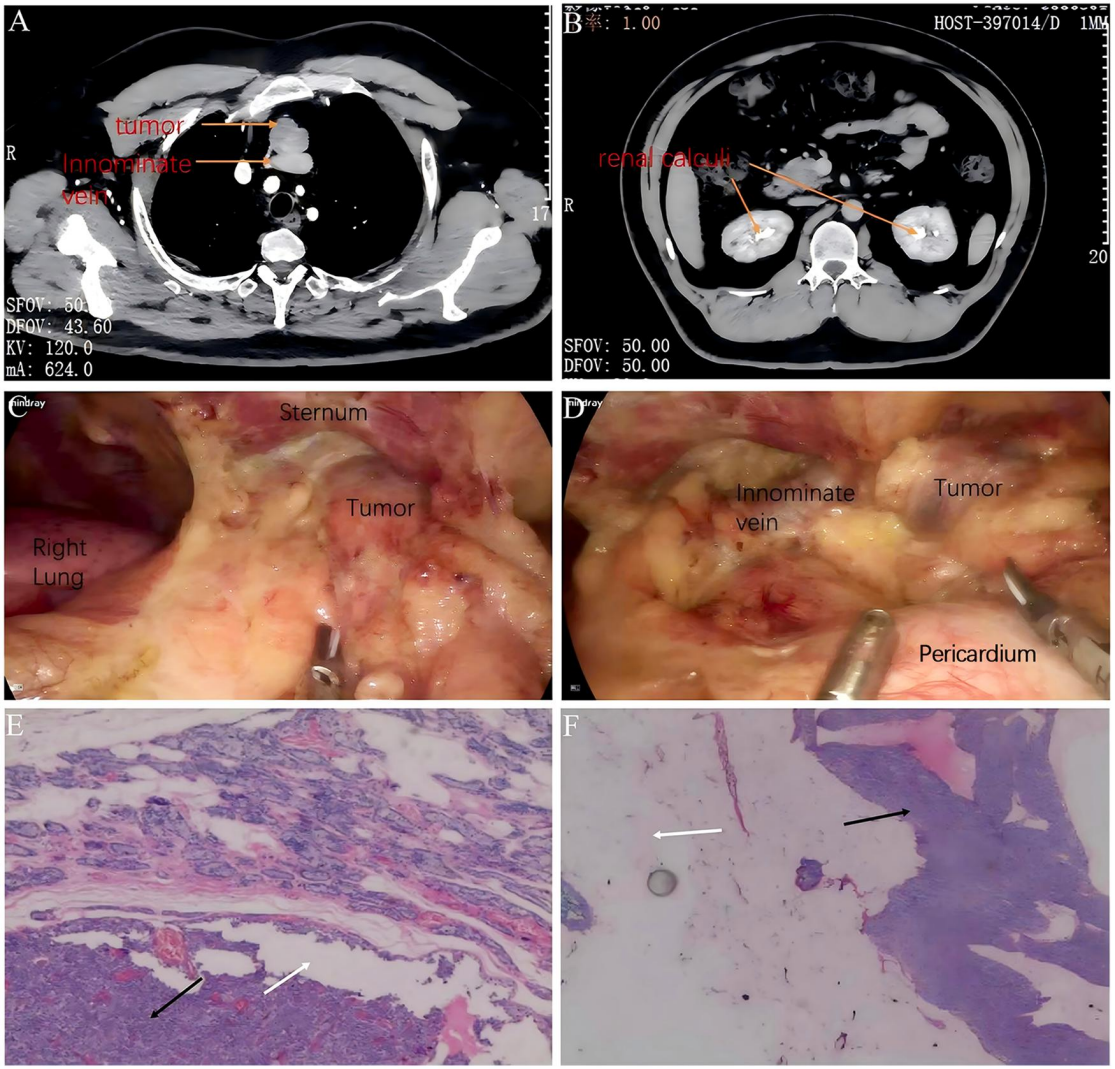
Preoperative imaging, intraoperative findings, and histopathological features of retrosternal parathyroid lipoadenoma. **(A, B)** Axial contrast-enhanced CT scan showing the high-lying tumor superior to the innominate vein and bilateral renal calculi. **(C, D)** Intraoperative screenshots obtained during subxiphoid VATS, showing the retracted tumor with the innominate vein (IV) visualized in the deep surgical field. **(E, F)** Hematoxylin and eosin (H&E) staining at 200× magnification of parathyroid lipoadenoma. Black arrows indicate nests of parathyroid chief cells, while white arrows highlight the abundant mature adipose stroma characteristic of this tumor.

Preoperative neck ultrasonography showed no abnormalities in the thyroid or parathyroid regions. A contrast-enhanced chest computed tomography (CT) scan identified a 30 × 27 mm nodular soft-tissue density lesion in the anterior superior mediastinum, located superior to the innominate vein. The mass showed heterogeneous enhancement with non-enhancing areas. Measurement of CT attenuation within the non-enhancing areas showed negative values ranging from −81.7 to −153 HU, confirming the presence of fat (Figures 1A,B). CT imaging revealed a mass within the anterior superior mediastinum that was highly suggestive of thymoma.

#### 2.1.2 Laboratory data (retrospectively analyzed)

Routine preoperative blood tests revealed significant albumin-corrected hypercalcemia (2.98 mmol/L, serum albumin: 46.5 g/L; reference: 40–55 g/L; reference for calcium: 2.2–2.7 mmol/L) and hypophosphatemia (0.8 mmol/L; reference: 0.85–1.51 mmol/L). Notwithstanding the presence of a mediastinal mass, recurrent nephrolithiasis, and definite hypercalcemia, no PTH level was measured preoperatively, and the diagnosis of primary hyperparathyroidism (PHPT) was not considered. In addition, no symptoms associated with hyperparathyroidism and no positive family history of the disease were documented upon admission.

### 2.2 Surgical strategy and technique

The patient's obesity, short neck, and the high anatomical position of the tumor rendered a transcervical approach unsafe. A decision was made to proceed with a transthoracic subxiphoid VATS approach to avoid median sternotomy.

Under general anesthesia with double-lung ventilation through single-lumen endotracheal intubation, the patient was placed in the supine position. A 3-cm vertical subxiphoid incision was made, and two additional operating ports were established under the bilateral costal arches. An artificial pneumothorax of approximately 10 mmHg was established to improve exposure of the narrow retrosternal space in this obese patient. During dissection, the bilateral phrenic nerves and brachiocephalic veins were carefully identified and preserved. The tumor, situated cephalad to the left innominate vein, was dissected using an ultrasonic knife and removed en bloc with the surrounding mediastinal fat (Figures 1C,D). The procedure was completed successfully without intraoperative complications.

### 2.3 Postoperative course and definitive diagnosis

#### 2.3.1 Postoperative day 1

The patient developed acute hypocalcemic tetany. Albumin-corrected serum calcium was critically low at 2.04 mmol/L (serum albumin: 41 g/L; reference: 40–55 g/L). The initial postoperative PTH level was 7.53 pg/mL (reference range: 15–65 pg/mL), a markedly low value indicating complete removal of the hormone-secreting adenoma.

#### 2.3.2 Postoperative day 5

The PTH level rose paradoxically to 125 pg/mL. The histopathological examination confirmed the diagnosis of a parathyroid lipoadenoma (Figures 1E,F), characterized by nests of chief cells within a stroma composed of more than 50% mature adipose tissue.

#### 2.3.3 Postoperative day 9

PTH levels peaked at 255 pg/mL, while serum calcium was 2.23 mmol/L (serum albumin: 39.4 g/L; reference: 40–55 g/L). The patient was managed aggressively with intravenous calcium gluconate, followed by oral calcium carbonate combined with cholecalciferol (each tablet containing 600 mg of elemental calcium and 125 IU of cholecalciferol, one tablet twice daily) plus calcitriol (0.25 μg once daily).

### 2.4 Follow-up

A ⁹⁹mTc-MIBI dual-phase scan with SPECT/CT performed 1 month postoperatively showed no evidence of residual or ectopic hyperfunctioning parathyroid tissue. The patient was followed for 10 months. His PTH level demonstrated a gradual declining trend, and his calcium and phosphate levels stabilized within the normal range without supplementation, confirming curative resection and restoration of metabolic homeostasis (Table 1).

**Table 1:** Dynamic postoperative changes of PTH and Serum calcium.

Date	Clinical event	PTH (pg/mL)	Albumin-corrected serum calcium (mmol/L)	Serum albumin (g/L)
3 November 2024	Preoperative	Not tested	2.98	46.5
7 November 2024	POD 1 (postoperative day 1), Tetany	7.53	2.04	41
11 November 2024	POD 5, Pathology Result	125.00	2.04	39.4
15 November 2024	POD 9	255.00	2.23	Not tested
7 December 2024-	Endocrine Follow-up	230.00	2.35	47.4
5 January 2025	Outpatient	82.20	2.36	44.4
18 September 2025	Last follow-up	72.10	2.36	46.5

##  Discussion

This case provides a platform to discuss the multifaceted diagnostic challenges of parathyroid lipoadenoma, emphasizing diagnostic pitfalls, evolving surgical strategies, and the pathophysiology of postoperative recovery.

### 3.1 The diagnostic pitfall: a classic cognitive error

The preoperative misdiagnosis in this case is a classic example of a clinical cognitive error.

The diagnostic challenge is 2-fold: The fatty component can lead to misinterpretation on imaging as a more common mediastinal tumor (e.g., thymoma or thymolipoma), and the ectopic location often renders standard cervical ultrasound non-revealing. Furthermore, the surgical approach to high-lying retrosternal tumors, particularly in obese patients, requires advanced minimally invasive techniques to avoid the morbidity associated with median sternotomy.

The failure to synthesize these findings underscores the need for heightened clinical vigilance. Parathyroid lipoadenoma poses a specific radiological challenge. While it is rich in fat, the intermixing with cellular components can result in a non-specific soft-tissue appearance on CT, as seen in our case, leading to its confusion with thymomas (2, 4). Functional imaging with ⁹⁹mTc-MIBI SPECT/CT is the cornerstone for localizing ectopic adenomas, with high sensitivity and specificity (5). Its use preoperatively could have been diagnostic, and its use postoperatively in our patient confirmed surgical cure.

### 3.2 Evolution of surgical approaches to retrosternal tumors

The management of mediastinal parathyroid adenomas has evolved from open sternotomy to minimally invasive techniques. VATS has been established as a safe and effective approach, associated with reduced morbidity, less pain, and shorter hospital stays compared to sternotomy (6). The subxiphoid approach, as utilized in our case, offers a superior midline view of the anterior mediastinum and is particularly advantageous for tumors in the high retrosternal position. It is especially beneficial for patients with an obese body habitus and short neck, where a cervical approach is suboptimal (7). Our successful resection, which required dissection around the great vessels and phrenic nerves, adds to the growing literature supporting subxiphoid VATS as a first-line option for complex anterior mediastinal pathology.

### 3.3 Postoperative biochemistry: biphasic PTH and hungry bone syndrome

The postoperative biochemical course in our patient is a textbook illustration of the metabolic changes following parathyroidectomy for severe PHPT. The initial plunge in PTH is a definitive marker of successful adenoma resection (8). The subsequent, often dramatic, rise in PTH is a physiological compensatory response, not indicative of failure. This “biphasic” pattern is driven by the combination of severe hypocalcemia from “Hungry Bone Syndrome” and the release of the remaining normal parathyroid glands from chronic suppression (9, 10). Hungry Bone Syndrome occurs due to the avid remineralization of the skeleton after the removal of the PTH-driven resorptive stimulus. Our patient's profound hypocalcemia requiring aggressive calcium supplementation occurred in the setting of a markedly elevated preoperative albumin-corrected serum calcium level (2.98 mmol/L; serum albumin: 46.5 g/L; reference: 40–55 g/L), with PTH not measured preoperatively. Combined with the characteristic biphasic changes in postoperative PTH, this suggested severe preoperative hyperparathyroidism with significant skeletal involvement. Recognizing this pattern is crucial to avoid misinterpreting the compensatory PTH rise as persistent disease.

This study has several limitations that must be acknowledged. First, preoperative PTH was not tested, so the diagnosis of Hungry Bone Syndrome remains presumptive and cannot be definitively confirmed. The clinical judgment was primarily based on the patient's severe postoperative hypocalcemia and the characteristic biphasic PTH dynamics observed after tumor resection. Second, baseline laboratory indicators such as serum vitamin D, alkaline phosphatase and magnesium were not routinely examined before surgery, further limiting the comprehensive assessment of the patient's preoperative endocrine and bone metabolic status. Third, the patient had a long history of recurrent nephrolithiasis with prior surgical intervention at another hospital; however, complete stone composition analysis and historical endocrine examination results were unavailable for preoperative clinical evaluation.

## 4 Conclusion

This case highlights the diagnosis and treatment of ectopic parathyroid lesions in the retrosternal mediastinum and offers important clinical reminders for clinicians. For patients presenting with a mediastinal mass complicated by hypercalcemia, clinicians should actively screen for ectopic primary hyperparathyroidism and avoid isolated interpretation of imaging findings alone. The subxiphoid VATS approach is a preferred surgical option for high retrosternal tumors in patients with unfavorable anatomical conditions such as obesity and short neck. In addition, recognition of the biphasic changes in postoperative PTH together with the clinical manifestations of Hungry Bone Syndrome enables clinicians to correctly interpret perioperative biochemical results and avoid misjudgment of treatment efficacy.

## Data Availability

The original contributions presented in the study are included in the article/Supplementary Material, further inquiries can be directed to the corresponding author.
